# A Pregnant Woman Who Underwent Laparoscopic Adrenalectomy due to Cushing's Syndrome

**DOI:** 10.1155/2014/283458

**Published:** 2014-12-03

**Authors:** Halit Diri, Fahri Bayram, Yasin Simsek, Yusuf Ozkan, Alper Akcan, Ibrahim Karahan, Ibrahim Ileri, Sulbiye Aribas, Mehmet Sait Koc

**Affiliations:** ^1^Division of Endocrinology, Erciyes University Medical School, 38039 Kayseri, Turkey; ^2^Division of Endocrinology, Firat University Medical School, 23000 Elazığ, Turkey; ^3^Department of General Surgery, Erciyes University Medical School, 38039 Kayseri, Turkey; ^4^Department of Radiology, Erciyes University Medical School, 38039 Kayseri, Turkey; ^5^Department of Internal Diseases, Erciyes University Medical School, 38039 Kayseri, Turkey

## Abstract

Cushing's syndrome (CS) may lead to severe maternal and fetal morbidities and even mortalities in pregnancy. However, pregnancy complicates the diagnosis and treatment of CS. This study describes a 26-year-old pregnant woman admitted with hypertension-induced headache. Hormonal analyses performed due to her cushingoid phenotype revealed a diagnosis of adrenocorticotropic hormone- (ACTH-) independent CS. MRI showed a 3.5 cm adenoma in her right adrenal gland. After preoperative metyrapone therapy, she underwent a successful unilateral laparoscopic adrenalectomy at 14-week gestation. Although she had a temporary postoperative adrenal insufficiency, hormonal analyses showed that she has been in remission since delivery. Findings in this patient, as well as those in previous patients, indicate that pregnancy is not an absolute contraindication for laparoscopic adrenalectomy. Rather, such surgery should be considered a safe and efficient treatment method for pregnant women with cortisol-secreting adrenal adenomas.

## 1. Introduction

Pregnancy is rare in premenopausal women with Cushing's syndrome (CS), because secretion of excess glucocorticoids inhibits the synthesis of gonadotropins, resulting in disorders in ovarian and endometrial functions, as well as amenorrhea or oligomenorrhea [1]. Furthermore, even if CS patients become pregnant, the incidence of serious complications is high. CS may cause maternal hypertension, diabetes mellitus/impaired glucose tolerance, osteopenia/osteoporosis, preeclampsia, pulmonary edema, heart failure, opportunistic infections, and even death [2–5]. In addition, CS may cause stillbirth, prematurity, and intrauterine growth restriction of the fetus [2–5]. Although it is crucial to recognize CS early in pregnancy, the diagnosis of CS may be overlooked due to the overlapping signs of preeclampsia and/or gestational diabetes.

In addition to its manifestations, including hypertension, hyperglycemia, and hypercortisolemia, the underlying etiology of CS must also be effectively treated in pregnant women. This underlying etiology includes adrenal adenomas, present in 15% of nonpregnant female patients with CS and in approximately 50% of pregnant women with this disorder [2, 3, 6]. Laparoscopic adrenalectomy is the first line treatment of choice in patients with cortisol-secreting adrenal adenomas, while medical treatment is suggested when surgery is contraindicated [7, 8]. Additionally, medical treatment should be administered preoperatively to decrease the likelihood of complications that occur due to adrenalectomy in patients with severe CS. Metyrapone is a preferred agent, because it has very rarely reported side effects in pregnancy. It may rarely exacerbate hypertension and thus cause preeclampsia [4, 9–11], and fetal hypoadrenalism may develop after metyrapone [12]. Ketoconazole is shown to cross placenta and can be teratogenic; thus it is in FDA category C [4].

Since CS is rarely encountered in pregnant women, laparoscopic adrenalectomy is rarely performed on pregnant women with adrenocorticotropic hormone- (ACTH-) independent CS. Here we describe a pregnant woman diagnosed with CS who underwent a successful unilateral laparoscopic adrenalectomy.

## 2. Case

A 26-year-old, gravid-2 para-1, 12-week pregnant woman was admitted to another hospital for headache and hypertension. A physical examination showed a body-mass index of 31.8 kg/m^2^, central obesity, hypertension (180/100 mmHg), a moon face, fascial plethora, and abdominal purple striae >1 cm wide. Hence, CS was suspected, and she was referred to Erciyes University Medical School.

**Figure 1:**
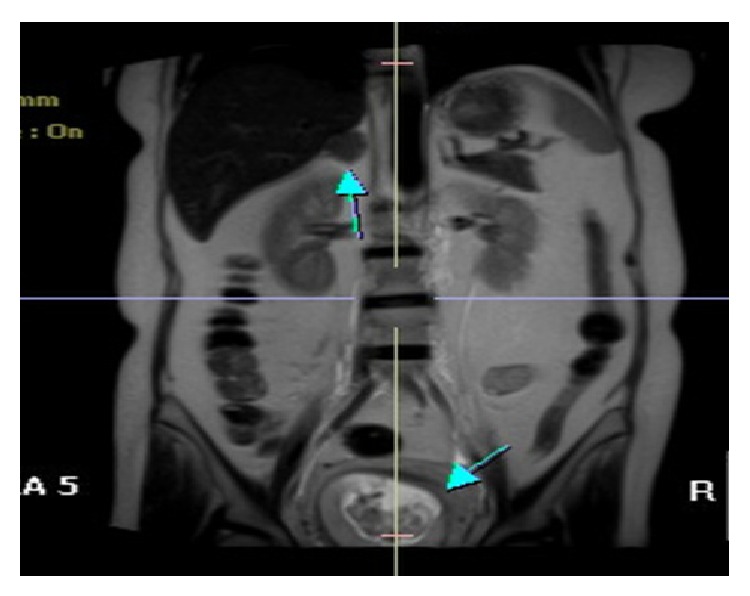
MR image of the adrenal adenoma and intrauterine fetus.

Firstly, she was treated with methyldopa to control her hypertension. Complete blood count and biochemistry tests including plasma glucose, liver and renal functions, and electrolytes were normal. Fetal ultrasound imaging was also normal and intrauterine growth restriction was not reported. Subsequent hormonal analyses showed a serum cortisol concentration of 48.2 *μ*g/dL after a 2 mg low dose-dexamethasone suppression test (LD-DST), a midnight serum cortisol concentration while awake of 32.0 *μ*g/dL, and a urine free cortisol (UFC) concentration of 2358 *μ*g/24 h (normal: 55–280 *μ*g/24 h). In addition, baseline serum cortisol and ACTH concentrations were 44.4 *μ*g/dL and 4.08 pg/mL, respectively, and serum cortisol concentration after 8 mg high dose-DST was 48.7 *μ*g/dL. The patient was diagnosed with ACTH-independent CS, and the diagnosis was confirmed by magnetic resonance imaging, which showed a 3.5 × 2.5 cm adenoma in her right adrenal gland (Figure 1).

The management of her hypertension became easier after metyrapone before the adrenalectomy, probably due to lower cortisol levels. After 6 days of metyrapone, the patient successfully underwent a unilateral laparoscopic adrenalectomy at 14-week gestation. The surgery was performed under general anesthesia; during the operation she was placed in the left lateral decubitus position. Carbon dioxide was used for insufflation of the peritoneal cavity to intra-abdominal pressure of 10 mmHg. No complications developed during surgery. Four days after surgery, her serum cortisol concentration, in a blood sample taken at 08:00 a.m., was 2.8 *μ*g/dL. The patient, who also had fatigue and anorexia, was administered 20 mg bid hydrocortisone replacement due to adrenal insufficiency. Since the patient did not have any medical problems and her hypertension had improved without drugs after surgery, she was discharged two weeks after the laparoscopic adrenalectomy.

The pathological examination of the removed adenoma was consistent with an adrenocortical adenoma. A follow-up visit performed 2 months after discharge and 2 days after discontinuation of hydrocortisone showed a serum cortisol concentration of 22.4 *μ*g/dL. Replacement therapy was therefore stopped and 1 week after, the cortisol level was 1.75 *μ*g/dL after 1 mg DST. She gave birth to a healthy baby at 41-week gestation by normal delivery. The baby's birth weight was 3.0 kg. Two and eight months after delivery, 1 mg DSTs showed cortisol concentrations of 1.05 and 1.40 *μ*g/dL, respectively, indicating that she remained in remission.

## 3. Discussion

The physiological increases in pituitary-adrenal axis activity during pregnancy cause serum ACTH, corticosteroid binding protein, total and free cortisol, and UFC concentrations to increase [4]. Therefore, pregnancy-induced physiological hypercortisolemia complicates the diagnosis of CS [13]. Measurements of midnight serum and salivary cortisol concentrations, 2 mg LD-DST, and UFC concentration are recommended for the diagnosis of CS in pregnant women suspected of having the disease [14–16], with positive results in two or more of these tests diagnostic for CS [17]. However, the threshold values may be higher in pregnant than in nonpregnant women. Because UFC increases throughout gestation, UFC concentrations 3-fold higher than the upper limit of normal for nonpregnant women should be regarded as the threshold during the second and third trimesters of pregnancy [18, 19]. Similarly, midnight salivary cortisol concentrations during the third trimester of pregnancy are twice as high as in nonpregnant women [20]. The likelihood of false-positive results on 1 mg DST is very high in pregnant women, making it logical to perform 2 mg LD-DST in pregnant women suspected of having CS. Serum ACTH concentrations are not suppressed in about half of pregnant women with cortisol-secreting adrenal adenoma due to placental CRH [4, 16]. In contrast, the circadian rhythm is preserved in healthy pregnant women [18, 21].

The combination of a typical cushingoid phenotype, very high cortisol concentrations, low basal ACTH concentration, and adrenal adenoma images on MRI led to a diagnosis of ACTH-independent CS in our patient. As these diagnostic analyses were performed during an early stage of pregnancy, the patient's serum ACTH concentration may have been suppressed by low levels of placental CRH.

It is not necessary to postpone the treatment of adrenal adenoma induced CS until after delivery because of concerns about complications of treatment methods [22, 23]. Due to the possible side effects of long-term medical therapy, such as hypertension and preeclampsia [9–11], surgery performed by an experienced team should be the first choice of treatment [7, 8]. Administering medical therapy until delivery may increase the likelihood of side effects and fetal problems such as hypoadrenalism [12]. On the other hand, it should be kept in mind that surgical complications may occur with a nonexperienced surgical team.

Surgery should preferentially be performed during the second trimester of pregnancy [8, 23, 24]. Although adrenalectomies performed during the third trimester have been reported to be successful [5, 6, 8, 25], administering medical therapy and delaying surgery until after delivery is more convenient in pregnant women diagnosed with CS during the last weeks of the third trimester [8].

Due to the risks of surgical complications during pregnancy, few pregnant women with adrenal CS have undergone adrenalectomy to date. Case reports, however, have indicated that the laparoscopic approach is safe, with the rates showing that maternal and fetal complications are higher in untreated than in surgically treated patients [26]. The major advantages of laparoscopic surgery are less intraoperative blood loss, less postoperative pain, more rapid wound healing, and earlier postoperative mobilization [27, 28]. Since the most frequently encountered surgical complication in pregnant women is persistent pneumoperitoneum after surgery, insufflation of the peritoneal cavity should not exceed 12 mmHg pressure [29].

Our findings indicate that pregnancy is not an absolute contraindication for laparoscopic adrenalectomy. In contrast, such surgery should be considered a safe and efficient treatment method for a pregnant woman with adrenal CS. Cortisol-secreting adrenal adenomas in pregnant women should be diagnosed and treated by experienced teams of related departments, with laparoscopic adrenalectomy regarded as the mainstay of treatment.
